# The Cost Effectiveness of Ecotherapy as a Healthcare Intervention, Separating the Wood from the Trees

**DOI:** 10.3390/ijerph182111599

**Published:** 2021-11-04

**Authors:** Sebastian Hinde, Laura Bojke, Peter Coventry

**Affiliations:** 1 Centre for Health Economics, University of York, York YO10 5DD, UK; laura.bojke@york.ac.uk; 2 Department of Health Sciences, University of York, York YO10 5DD, UK; peter.coventry@york.ac.uk; 3 York Environmental Sustainability Institute (YESI), University of York, York YO10 5NG, UK

**Keywords:** ecotherapy, mental health, physical health, greenspace, nature-based intervention, economic evaluation, cost-effective analysis

## Abstract

Internationally, shifts to more urbanised populations, and resultant reductions in engagements with nature, have been a contributing factor to the mental health crisis facing many developed and developing countries. While the COVID-19 pandemic reinforced recent trends in many countries to give access to green spaces more weight in political decision making, nature-based activities as a form of intervention for those with mental health problems constitute a very small part of patient pathways of care. Nature-based interventions, such as ecotherapy, are increasingly used as therapeutic solutions for people with common mental health problems. However, there is little data about the potential costs and benefits of ecotherapy, making it difficult to offer robust assessments of its cost-effectiveness. This paper explores the capacity for ecotherapy to be cost-effective as a healthcare intervention. Using a pragmatic scoping review of the literature to understand where the potential costs and health benefit lie, we applied value of information methodology to identify what research is needed to inform future cost-effectiveness assessments. We show that there is the potential for ecotherapy for people with mild to moderate common mental health problems to be cost-effective but significant further research is required. Furthermore, nature-based interventions such as ecotherapy also confer potential social and wider returns on investment, strengthening the case for further research to better inform robust commissioning.

## 1. Introduction

The mental health crisis faced by the UK and internationally is well evidenced, with an estimated 1 in 4 adults in the UK experiencing some form of mental health problem in any given year [1]. Mental health problems are estimated to now cost the National Health Service (NHS) in England £14 billion annually [2], and the wider economy up to £100 billion [3], in addition to the enormous human suffering they entail [4]. The prevalence of mental health problems has at least doubled during the COVID-19pandemic [5], but there has been a reduction in help-seeking and identification of mental health problems in primary care and mental health services [6]. While during the pandemic access to green space has been seen as an important asset to promote wellbeing and prevent mental ill health [7], this is against a backdrop of many individuals not considering exposure to natural environments to be an important part of their lives [8] and a global shift to more urbanised populations [9], and an associated reduction in exposure to green spaces [10].

The role of engagement with nature as a means of promoting good mental health is nothing new, with forms of active or passive engagement predating many more modern approaches [11]. The causal relationship between exposure to nature and mental health has also been explored in much of the literature [10,11], with research beginning to demonstrate the benefits of some nature-based interventions on depression and anxiety through randomised control trials [12]. More broadly, less robust evidence is emerging on the impact of exposure to nature on social and economic factors including employment, disability allowance claims, tax receipts, out of pocket costs of illness, inequality and health burden [13]. These benefits are achieved through the wide range of interactions that are facilitated by and intrinsic to exposure to nature, including physical activity, socialising and mindfulness [11]. However, there has been a push to identify and quantify health benefits of nature in more robust ways to better inform policy and commissioning decisions.

Ecotherapy is one form of active healthcare intervention which attempts to draw on the benefits of nature-based activity to support people with mental health problems. It can be broadly defined as a facilitated therapeutic intervention based in a natural setting, typically done in a group, with a focus on the activity rather than the participant’s health [14]. Examples include walking groups, bushcraft skills and creative writing. The treatment modality is described elsewhere [11] but in brief as a set of interventions they draw on known evolutionary, physiological and social approaches to improving mental health, aiming to achieve them through activities intrinsic to natural exposure, including physical activity, socialising and mindfulness. Consistent with a recent systematic review about the effectiveness of nature-based interventions we distinguish ecotherapy from nature-based interventions that are neither group based nor facilitated by a trained therapeutic coordinator [12]. Examples of such may include gardening, conservation or creative tasks but importantly missing the key features of being conducted in a facilitated group.

However, in the UK, despite recent reviews by the UK Government’s public health body [13] and some funding for recent Green Prescribing policies [15], nature based mental health services such as ecotherapy still rely predominantly on charitable and piecemeal local funding, rather than any national commitment [16]. This has resulted in the set-up and delivery of over 1000 projects which provide support for 21,000 individuals in England alone. However these services are unevenly distributed across a geographically diverse area, leading to inequalities in service provision and little scrutiny of their quality and effectiveness [16].

The inconsistent availability of services is compounded by a lack of high-quality research quantifying the benefits of nature based mental health services either from a clinical or cost-effectiveness standpoint [12,16,17,18]. This results in limited evidence available to commissioners with which to conduct assessments of value consistent with good methods guidance [19], and therefore limited grounds for support.

If the long-term sustainable funding of potentially cost-effective nature-based services such as ecotherapy is to be secured, a clearer narrative is required regarding the value proposition to commissioners. To demonstrate the value of commissioning provision and research into services such as ecotherapy, this paper will explore the mechanisms by which ecotherapy (and similar interventions) potentially generate health and non-health gains, the evidence that still needs to be generated to quantify these gains, and by extension interrogate whether such interventions could ever be cost-effective.

The aim of this approach is to present the potential merits of further research into ecotherapy in order to prompt more robust assessment of the cost-effectiveness of nature-based interventions. To do so the paper draws on existing evidence of the indicative costs and benefits of ecotherapy in both the peer-reviewed and grey literature, identified through a scoping review. The findings of this review are then considered against a value of information framework, whereby the scale of uncertainty of the evidence around the costs and benefits of an intervention are contrasted with the value of resolving it with additional research. Further interpretation of our findings are drawn from the wider literature on mental health and nature-based interventions.

## 2. Methods

Fundamental to an assessment of value in any setting is the exploration of the beneficial effects of an intervention relative to its costs. In an English healthcare setting, supporting the funding of ecotherapy as a mental health intervention would require some indication of cost-effectiveness. Such evidence forms one element in the deliberations of the National Institute for Health and Care Excellence (NICE) in their commissioning recommendations and clinical guidance, as well as informing local commissioning decisions and value assessments [20].

In the NICE framework it is accepted that the decision whether to fund an intervention should be based on the expected incremental cost-effectiveness rather than the uncertainty around the decision [21]. However, the certainty around the commissioning decision still has value to the decision maker, with the presence of uncertainty risking an incorrect decision being made. Value of information (VoI) methodology seeks to estimate the value of resolving this uncertainty and therefore the value of conducting additional research after a commissioning decision has been made, either positively or negatively [22]. The use of these methods is common in the analysis of pharmaceuticals and interventions where the evidence is sufficiently mature to inform a robust decision analytic model, facilitating the estimation of specific outputs such as the expected value of perfect information. However, as a method it can more broadly be applied in areas where the evidence is still under developed, such as nature-based interventions for common mental health problems.

By using VoI methodology in such a setting it is possible to highlight not only whether ecotherapy has a reasonable likelihood of being cost-effective given current evidence but also identify where uncertainty has the largest impact on the decision problem, and therefore inform future research decisions [23]. This is in contrast to the conventional approach to generating evidence, where research is conducted often with little consideration of the long-term cost-effective consequences, but a focus on short-term clinical effectiveness alone [24].

We use the approach outlined by NICE in their cost-effectiveness reference case [25] to explore the potential value of ecotherapy, and by extension future research, as a purely health-based intervention, funded by the public healthcare system (i.e., the NHS in England) and positioned as a competing or complementary therapy to existing services for patients with common mental health problems, such as depression and anxiety.

A value assessment of ecotherapy using the NICE framework [25] can be condensed into a consideration of the cost and health impact of changing from the current NHS and Personal Social Services (PSS) care provision to the proposed intervention, measured over the lifetime of the patient. For the purposes of the NICE framework, all costs that fall outside of the NHS and social care budget, and benefits beyond the health of the patient/participant, within their lifetime, are considered outside of the healthcare remit. This approach is consistent with previous evaluations of interventions to improve mental health by NICE, including that for cognitive behavioural therapy (CBT) [26]. In their assessment of the benefit of an intervention the NICE framework primarily uses a measure of health-related quality of life that also reflects length of life, which is captured by the quality adjusted life year (QALY) approach. Details of the QALY approach are available elsewhere [27] but in brief it employs a generic measure of quality of life, most typically the EuroQol EQ-5D scale [28] used to weight the time spent in each health state, for example ill or cured.

We break this value assessment of ecotherapy as a health-based intervention into five component parts. In each case we summarise the results of the scoping review relevant to each section, and briefly describe the significance of each element to the assessment of value in terms of cost-effectiveness. Where appropriate additional exploratory analysis is conducted to estimate the cost or QALY impacts in terms of the NICE reference case. The five component parts are:

(1) Establishing the mental health benefits generated whilst being actively ‘treated’, i.e., engaging in the ecotherapy programme.
(2) Establishing the physical health benefit generated whilst being actively ‘treated’.
(3) Establishing how these shorter term mental and physical health benefits translate into longer term health gains.
(4) Establishing the cost of providing ecotherapy.
(5) Establishing the resource use impact to the NHS and PSS.

Upon summarising the results of the scoping review in these five elements we present an extensive discussion section which explores the potential meaning of the findings in terms of an overall cost-effectiveness assessment of ecotherapy. To reflect the broader benefits and cost profile associated with ecotherapy in the discussion we then explore the evidence of the impact of ecotherapy which would fall outside of the NICE reference case, and therefore potentially be excluded from an assessment of value and costs to the commissioner but represent important components in a societal discussion of the value of such programmes.

To guide the discussion of the potential scope and scale of the costs and health impact of ecotherapy in each of these component sections we conducted a pragmatic scoping review of the existing literature. A scoping approach was taken for several reasons. Firstly, a recent systematic review of nature based outdoor activities for mental and physical health [12] identified an insufficient pool of peer-reviewed evidence on the effectiveness of ecotherapy with which to inform this analysis in isolation. Secondly, due to the charitable funding of existing ecotherapy services much of the literature is ‘grey literature’, typically non-peer reviewed analysis commissioned by the charities. Finally, the exploratory nature of this analysis allows for the inclusion of a lower quality of evidence from studies that would not be sufficient to inform an economic evaluation aimed at directly informing policy. This includes case studies, many of which do not appear in peer-reviewed journals or bibliographic databases and are typically excluded from more formal systematic reviews.

The pragmatic search was conducted to identify studies of any design or quality which could be used to inform any of the five components of the exploratory cost-effectiveness analysis. The search was conducted in August 2020, initiated through an exploration of studies known to the authors and through searching for studies by using key words in an internet search engine. Using these papers bidirectional citation searching to completion, or ‘pearl growing’, was conducted [29]. Under this approach, the references and citations of key papers are used to identify additional literature which is then subsequently searched.

The inclusion criteria were defined as the reporting of novel evidence which could be used to inform any of the component parts of value assessment. In addition, to maximise generalisability to the UK context we only included studies that reported the use of interventions that mapped to ecotherapy and targeted adult populations with common mental health problems such as depression and anxiety. Due to the pragmatic nature of the search only relevant studies were recorded. While studies were not excluded based on their perceived quality of likelihood of bias, the quality of the studies was recorded through their size, data collection method and use of a control population.

## 3. Results

The pragmatic search identified six studies which met the inclusion criteria for the analysis, consisting of eight separate publications, these are detailed in Table 1. The applications of these studies to the five components of ecotherapy evaluated as a health-based intervention are explored in turn.

As indicated from the prior systematic reviews in the area [12,16,17,18] the quality of the studies was found to be poor. Half of the studies identified (*n* = 3) had no peer-review element in the analyses reported, with only two studies including any form of control group. Furthermore, the number of participants in the studies was often small with a lack of clarity regarding how participants were selected, including case studies of a handful of selected individuals in the case of the New Economic Forum analysis.

### 3.1. Mental Health Benefit during Delivery of the Intervention

Ecotherapy is typically characterised as a nature-based intervention that includes a range of outdoor activities with a primary focus on mental health improvement [17]. Natural England have argued that such interventions improve mental health by: connecting people to nature; facilitating social contact and support; and engaging people in tasks they regard as purposeful [16].

In general terms there is a known protective and restorative impact of green exposure and physical activity to mental health [38,39], and policy support for maintained and increase public access [13]. Similarly, ecotherapy interventions aiming to increase such exposure have shown a beneficial effect, albeit as small, uncontrolled studies. For example, Rogerson et al. (whose analysis drew data from a number of Wildlife Trust ecotherapy programmes) looked at the impact of six small exercise-based facilitated green interventions, finding that 61% of participants who started in a lower wellbeing state, using WEMWBS, moved to an average-high category [34].

Evidence from other ecotherapy interventions is limited and concentrated in non-peer reviewed studies conducted by funders of the services. However, these reports have indicated a potentially beneficial impact of ecotherapy on mental health factors. For example, Mind, a mental health charity in England who run several ecotherapy programmes, reported that 71% of participants in their guided green walk ecotherapy programme experienced a decrease in their depressive symptoms [32].

While previous research about the effectiveness of ecotherapy for mental health has not collected data required to directly inform a QALY impact, from a conceptual approach we can consider how the reported impact on patients’ depression would translate to the EQ-5D-5L. This conceptual approach allows us to consider what may be a feasible impact of ecotherapy during the period of the programme.

One of the five dimensions of the EQ-5D measures anxiety and depression. If we consider this dimension alone, keeping all other elements of the questionnaire fixed, we can identify that if a patient moves from reporting ‘I am moderately anxious or depressed’ (score 3 out of 5) to ‘I am slightly anxious or depressed’ (score 2) the estimated quality of life gain would be 0.015, equivalent to 0.015 QALYs if the change occurs for a full year. Similarly, for a more severe patient, a change from ‘I am severely anxious or depressed’ (score 4) to ‘I am moderately anxious or depressed’ (score 3) would imply a 0.213 quality of life gain for as long as this effect was maintained, as the uplift from the more extreme states on the scale is given a much greater weight in EQ-5D.

This change in quality of life score can be considered against the 61% shift from a low to average-high WEMWBS category reported in Rogerson et al. [34] to speculate that an ecotherapy programme could be associated with an improvement in patient reported quality of life of between 0.01 and 0.13 on the EQ-5D-5L scale (0.015 and 0.213, respectively, multiplied by 61%).

However, the literature does indicate that there is existing uncertainty regarding a positive impact on mental health. For example, Wilson [37] found no statistically significant impact of the Branching Out ecotherapy programme on mental (measured using WEMWBS) or general health (SF-12 questionnaire). They suggest that the lack of an effect is either due to too short a programme, or a ceiling effect due to patients already being engaged with mental health services. However, it is possible that they indicate a poor level of effectiveness of ecotherapy programmes to impact mental health outcomes, but it is not possible to determine which is the case without a well-designed trial. Furthermore, the evaluation of a series of interventions in woodlands in Scotland by Thompson et al. [36], including social activities, indicated an unexpected potential for increased stress with greater green space use, coupled with no quality of life impact.

### 3.2. Physical Health Benefit during the Programme

While ecotherapy is not typically offered as an exercise intervention, many forms of ecotherapy offer the means for participants to undertake some form of green exercise, and physical activity is an important secondary outcome and possible mediator of mental health outcomes [40]. Bagnall (as part of the Wildlife Trust analysis) reported that of the 19 patients who indicated a low baseline wellbeing score, 15 reported an increase in physical activity during their programme [35]. Similarly, the Mind evaluation found that 90% of those surveyed reported a beneficial impact on their physical health [32]. While Wilson found no beneficial impact of their ecotherapy on mental health outcomes, they did identify an average post-intervention increase of over 4 h in the amount of moderate to high intensity exercise of their participants, including the 3 h of their intervention [37].

Translating these sorts of gains in physical activity into a QALY gain is difficult, even if quantitative outcomes are recorded, as generic health outcome measures, such as EQ-5D, only indirectly measure physical activity by assessing mobility.

### 3.3. Health Benefit into the Medium and Long-Term

A key component of the cost-effectiveness of any intervention is its ability to affect long-term change through durable behaviour change. Fundamentally, in the case of ecotherapy, if the change in mental health only lasts for the duration of the programme the chance of cost-effectiveness is limited as the total QALY gain will be small compared with the lifetime of the patient.

Depression can be a long-term and recurrent illness and so treatment strategies that are likely to be effective over the long-term necessarily aim to put in place strategies that reinforce positive behaviours and build skills and confidence in self-management. For example, CBT includes components that give people skills to self-manage their mood and has been shown to be clinically and cost-effective over the long term when used as an adjunct to antidepressants [41]. Similarity, ecotherapy also offers participants opportunities to develop skills and the confidence to self-manage their health but there is an absence of evidence about long term mental and physical health benefits [18].

Unfortunately, we have been unable to identify any evidence directly reporting the impact of ecotherapy on long-term patient health, with all the studies identified in the scoping review only collecting evidence up to the end of the programme itself, typically a few months long.

### 3.4. The Cost of Providing Ecotherapy

Conventionally in a healthcare setting, the question of the cost of a new treatment is reasonably simple to assess. The cost of the new drug or technology is set by the manufacturer, and the costs of any required support systems, e.g., nurse time, calculated using standardised estimates of unit costs [42]. However, costing interventions such as ecotherapy is more complex, as the details of the intervention and market value are by their nature hard to determine due to the voluntary nature of much of what is provided in many current programmes.

Additional to the challenges which occur from costing existing programmes is the understanding of what such costs would mean for the estimation of the cost of expanding ecotherapy to become more nationally available. Following the NICE framework again, the relevant cost considered by an economic evaluation of an intervention is the marginal cost, i.e., the cost of a change in the level of the intervention provided, in contrast to the average cost. However, intrinsic to the cost of rolling out largescale ecotherapy programmes is the requirement for ecotherapy providers to have access to suitable green spaces. Currently, ecotherapy exists in relatively small-scale settings, such as care farms, nature reserves and public woodlands, venues that, where they exist, imply only small additional cost of providing the service compared to the cost that would be needed to set up an entirely new infrastructure where none currently exists. As a result, any existing cost evidence must be interpreted carefully, with an awareness of the challenges of disentangling factors as fixed and variable costs which would be important considerations to commissioners if such programmes were to be made widely available.

Through the pragmatic scoping review of the published and grey literature, and personal contact with existing providers, we were able to identify three estimates of the average cost of running an ecotherapy programme, each with its own assumptions and caveats. Mind’s 2013 evaluation of an ecotherapy programme reported a cost of £5400 to support a single person for a year at one of their care farms [33]. Additionally, we were able to identify two other estimates of costs though communication with two ecotherapy programmes, both anonymous for this analysis, one for elderly patients with dementia and one for general mental health problems. Per patient per year these costed £2340 and £636, respectively, with most of this cost attributed to staff costs. This significant variation in cost is likely due to not only variation in the programmes offered, but also in what costs are borne by each setting, and which are provided from other contributors or voluntarily resourced.

### 3.5. The Resource Use and Cost Impact of Ecotherapy

An intervention which improves the health of an individual will also be expected to reduce their healthcare needs over an equivalent time-period, resources which can then be allocated to the treatment of other patients. Therefore, in addition to the upfront cost of providing the service, the resource use profile of the individual over the long-term plays an important role in the assessment of cost-effectiveness.

Many of the potential cost savings that occur in the successful treatment of patients with common mental health concerns occur as a result of reductions in direct treatment needs, i.e., due to no longer needing long term pharmacological or clinical care. Currently, between £2 and £3 billion is spent every year by the NHS in England treating depression [43], with the average patient in contact with NHS mental health services costing over £2000. This issue is compounded by the long-term burden of common conditions such as depression, with almost one million adults in the UK estimated to be on anti-depressants continuously for at least three years [44].

Several ecotherapy studies point to the potential cost savings associated with reduced medication use and healthcare interactions, such as GP and mental health nurse consultations. The New Economic Forum’s evaluation of the Ecomind programme used these cost savings as the primary outcome of their analysis of five individual case studies [31]. While case-study analysis is of limited value to inform a full cohort analysis, it does provide insight into the potential maximum propensity to benefit. In one case the report indicates a potential NHS saving of £7635 for the year the intervention is active for a single participant, based on reductions in medication and previous high dependency on mental health support services.

Similarly, the return on investment analysis produced by City Farms and Community Gardens identified that among their 16 volunteers an annual total of 311 fewer visits to doctors occurred and 572 fewer visits to support workers, alongside three of the volunteers reducing their antidepressant use [30]. However, they did report that two volunteers also increased their antidepressant medication and noted that without a control group it was not possible to determine if the changes in NHS interaction were the result of the activities provided.

## 4. Discussion

### 4.1. Reflecting All Health Gains Using a Cost-Effectiveness Framework

As described earlier, under a NICE evaluative perspective the cost-effectiveness of an intervention such as ecotherapy is determined by combining the mental and physical health impact to the participant and the cost borne by the NHS and social service sectors, relative to existing care, into a single incremental cost-effectiveness ratio (ICER). This ICER is then compared to a notional threshold value to determine whether the additional cost burden is worth the health benefit. Therefore, to inform a decision on the cost-effectiveness of ecotherapy NICE, and other health technology assessment agencies, would seek to combine the elements covered by the previous five sections.

As reflected in published reviews in the area [12,16,17,18], the existing quality of evidence on the effectiveness and cost-effectiveness of ecotherapy and other nature-based interventions would be insufficient for a national health technology assessment agency, such as NICE, to conduct a meaningful assessment, reflecting why it has not been included in the relevant clinical guidance [26]. However, that is not to say that no indicative evidence can be drawn from the existing literature which can be combined and used to inform current commissioning policy and future research through value of information methodology.

The existing evidence relevant to the cost-effectiveness of ecotherapy for the treatment of depression, taking a conventional NICE perspective, appears to indicate that there is the potential for mental and physical quality of life benefits during the period of the intervention, as well as cost savings from reductions in demand for existing care services and medication. However, this evidence is of poor quality and limited scale, and there is no directly relevant evidence regarding the long-term efficacy of ecotherapy. The lack of long-term efficacy data is key as the relatively high cost of providing ecotherapy, both in terms of initial investment in infrastructure and running costs, suggests that even a substantial QALY benefit during the period of ecotherapy engagement may not be sufficient to justify cost-effectiveness from a NICE perspective.

### 4.2. A Broader Perspective on Value

Arguably, one of the main appeals of ecotherapy as an intervention is that it can yield benefits that go beyond health [13]. In this sense the NHS perspective represents a small proportion of the total potential benefit to stakeholders, evidenced by studies such as the City Farms and Community Gardens social return on investment (SROI) analysis estimating a social return of £3.56 for each pound invested in the Gorgie City Farm project, only a small fraction of which were returns realised by the NHS [30]. A range of potential wider benefits of ecotherapy have been proposed and reported, including employment, disability allowance claims, tax receipts, national insurance contributions, out of pocket costs of illness, benefits to carers, benefits to wider society of investment in green spaces, impact on inequality, loneliness, crime levels and environmental benefits [13].

However, the current NICE framework which informs the assessment of value detailed so far in this paper, while acknowledging impacts outside the NHS and QALY-based definition of health, does not currently incorporate any wider elements into the estimation of cost-effectiveness of an NHS funded intervention [25]. As a result, while methods such as SROI have been proposed and applied in this setting [13], they do not represent a methodological approach that is considered to be sufficient in the justification of cost-effectiveness from an NHS perspective, primarily as many of the costs and benefits included fall outside what is considered relevant to NHS commissioning. While methods are beginning to emerge that allow the use of a wider definition of impact into the current NICE framework [45], these are yet to be applied to the ecotherapy setting or routinely in commissioning decisions, and ultimately still require significant deliberation and agreement across different decision-making groups.

### 4.3. Transferable Findings from Other Interventions and Settings

Beyond ecotherapy, previous studies have sought to estimate the value of changes in activity to patient health and NHS costs to facilitate the use of short-term estimates of change in physical activity such as those indicated in much of the ecotherapy literature. For example, the analysis conducted alongside NICE’s current physical activity guideline found that an intervention that cost £1 million could be cost effective from a NICE perspective if it encouraged 1900 people to walk an extra 30 min per week long term [46]. This analysis was premised on an understanding that increased physical activity would reduce the risk of health problems such as cardiovascular disease.

More broadly Natural England estimated that £2.1 billion could be saved in healthcare costs if everyone had access to green spaces, due to the beneficial impact on mental health and physical fitness [47]. Similar estimates include £760 million in avoidable medical costs if people had one or more ‘active’ visits per week to a greenspace [48], and a return of investment of £1:£34 for spending on park maintenance to healthcare cost saving [49].

The estimates that we present of the financial benefits that could be achieve from ecotherapy and green space exposure need to be interpreted carefully. In addition to the issues of the design of the underlying trials and case studies, the estimations of unit costs are highly uncertain and subject to many assumptions. However, as with the claims for the longer-term health benefit of ecotherapy, they do indicate a potential cost saving that would merit further exploration using more robust methodology such as a randomised controlled trial.

Similarly, there is extensive literature, largely from the United States, regarding the use of other nature-based therapeutic methods including wilderness therapy and outdoor behavioural healthcare. While the evidence on such interventions is more mature than ecotherapy, for example studies by Gass et al. [50] and Kuo and Faber-Taylor [51], questions remain about the comparability of the patient populations studied, with these typically focussing on children with addiction or behavioural issues, and the transferability of studies between countries.

### 4.4. The Value of Additional Information Regarding the Cost-Effectiveness of Ecotherapy

There is potential for nature-based interventions, such as ecotherapy, to provide treatment and support for the growing population of people with mental health problems internationally. This is reflected in the broad range of literature on the value of green space exposure [18] as well as recent UK Government support for green social prescribing [15]. However, as Summers and Vivian reflected [18], nature based interventions are very difficult to evaluate due to challenges of demonstrating a causal link and ensuring sufficient intervention control on a fundamentally holistic intervention.

As a relatively structured intervention compared to general funding of green spaces, and which has been shown to be a viable compliment to existing publicly provided mental health provision [40], ecotherapy has the potential to bridge the gap between the rigorous requirements of evidence generation to inform commissioning and the holistic nature of nature based investments. However, its current lack of universal availability or NHS funding pathway, makes it hard to make policy and commissioning decisions given the existing evidence base. A randomised control trial of an ecotherapy programme, implemented following best guidance on the evaluation of a health-based intervention and incorporating modelling of the potential cost-effectiveness of the NHS funding such a service, would represent a valuable step forward. Such a trial and evaluation would not only provide valuable evidence as to whether ecotherapy programmes have the potential to be effective and cost-effective as health interventions, but also provide a basis on which to build a discussion of the relative weight of the other costs and outcomes associated with such nature-based interventions.

This is not to acknowledge the significant challenges facing the generation of robust evidence for ecotherapy programmes. In addition to those intrinsic to nature based interventions [18] there are challenges related to the practicality of setting up programmes nationally such as replicability of ecotherapy programmes, the reliance on pre-existing natural infrastructure, as well as technically including the limitations of current methods of economic evaluation to fully incorporate the costs and benefits in a consistent way [52]. Some of these issues can be overcome through robust data collection and trial methodology, but others may not. For example, some outcomes of interest may never be accurately attributable to quantifiable outcomes due to issues of lags and complex dynamics.

Additionally, care will be needed to ensure the commissioning of nature-based interventions does not exacerbate the existing socio-economic divide in both individual’s ability to access green spaces and the burden of mental and physical illness.

## 5. Conclusions

Current availability of ecotherapy in the UK is reminiscent of the provision of specialised pharmaceuticals by the NHS prior to the inception of the NICE technology appraisal process in 1999. One the of the leading justifications for the creation of NICE was to do away with the ‘postcode lottery’ of drug availability, where the commissioning of many high-cost medications was decided locally and was argued to undermine the equity principles of the NHS. Similarly, ecotherapy is only available in a limited number of areas with the lack of robust evidence or national guidance undermining the ability to achieve consistent commissioning practice. While the NICE process has many limitations, primarily the tight definitions of whose costs and outcomes matter in their main estimation of effectiveness and cost-effectiveness, it provides a valuable basis from which to have transparent and importantly consistent discussions about such limitations. Currently, the lack of robust evidence on the role of ecotherapy as a funded health intervention has meant that there are insufficient grounds from which to base such a discussion. A randomised control trial with sufficient statistical power and follow-up is urgently needed if the evidence is to catch up with the current national policy enthusiasm for nature-based interventions.

## Figures and Tables

**Table 1:** Table of studies that conducted primary data collection relevant to any of the five components.

Study	Publication Type	Study Design	Population	Intervention	Control	Outcome	Headline Result
Pank et al. [30]	Commissioned report, not peer-reviewed.	Survey, social return on investment analysis, and qualitative study.	Broad range of stakeholders of the Gorgie City Farm.	Engagement with (as volunteer or visitor) Gorgie City Farm.	None	Broad range of outcomes related to mental health, eating habits, education, environment, NHS interactions.	Estimated a SROI of £3.56 for social value for every £1 invested in the project.
New Economic Forum [31]	Commissioned report, not peer-reviewed.	Case reports and costing analysis.	5 selected Ecominds participants.	5 different Mind funded Ecominds projects.	None	Avoided public sector costs.	Annual public sector costs avoided of between £4151 and £12,799 per person.
MIND [32,33]	Commissioned report containing two studies, not peer-reviewed.	Survey	Unspecified members of a local Mind green exercise groups.	19 groups covering a range of gardening, conservation, and walking activities.	None	Qualitative questionnaire related to the benefits of the groups.	109 questionnaires returned showing potential benefits of nature-based activities.
		Survey	20 members of local Mind associations (age 31 to 70).	Half an hour walk in a country park.	Half an hour walk inside a shopping centre.	Questionnaire covering self-esteem, mood, and mood disturbance.	Outside walk showed increase in all aspects of self-esteem and mood.
Wildlife Trust [34,35]	Unfunded peer-reviewed publication.	Before and after study.	318 members of 6 existing nature-based wellbeing projects.	Interventions range in design but all nature-based with focus on mental and physical wellbeing.	None	WEMWBS wellbeing scale collected at the start and end of each project.	Pooled results showed large improvement in wellbeing.
Thompson et al. [36]	NIHR funded peer-reviewed publication.	Before and after controlled quasi-experimental study and cost-effectiveness analysis.	Community living near woodlands selected for improvement.	Physical improvements to woodlands and community engagement activities.	Control population selected who lived further from the selected woods.	Primary outcome was stress (using the PSS), secondary included EQ-5D, and physical activity.	2117 responses indicated intervention was associated with increases stress, with no change in EQ-5D or physical activity.
Wilson et al. [37]	Peer-reviewed publication.	Before and after study.	People referred from secondary and tertiary mental healthcare services.	12 week programme of multiple ecotherapy activities in two areas.	None	Mental (WEMWBS), physical (SPAQ) and general (SF12) health questionnaires.	Little change in mental or general health but significant increase in physical activity.
